# Prospective and dichotomous study of biomarkers with swept-source OCT and OCT-angiography in naive patients with diabetic macular edema

**DOI:** 10.1186/s40942-025-00672-7

**Published:** 2025-04-22

**Authors:** Marcussi Palata Rezende, Fernanda Atoui Faria, Daniel Prado Beraldo, Julia Polido, Rubens Belfort, Thiago Cabral

**Affiliations:** 1 Department of Ophthalmology, Federal University of São Paulo (UNIFESP), São Paulo, SP Brazil; 2 Clinica Oftalmo-Retina, Presidente Prudente, SP Brazil; 3 Department of Specialized Medicine, CCS and Vision Center Unit, Ophthalmology, EBSERH/HUCAM, CCS-UFES-Federal University of Espírito Santo (UFES), Vitoria, ES Brazil; 4 Rua: Engenheiro Alfred Johann Liemert, 237, Sala 5A, Presidente Prudente, SP CEP:19.061-251 Brazil

**Keywords:** Aflibercept, Swept-source OCT, OCT angiography, Diabetic macular edema, Diabetic retinopathy, Choroidal thickness, Biomarkers

## Abstract

**Background:**

We used state-of-the-art high-resolution retinal imaging to explore the treatment (loading dose of aflibercept) of diabetic macular edema (DME) among treatment-naive patients. Swept-source (SS) OCT and OCT-Angiography (SS-OCTA) were performed, and a dichotomous analysis was conducted to compare responders and treatment-resistant patients (responsive and resistant). Furthermore, treatment responses were evaluated based on the subdivision of choroidal thickness.

**Materials and methods:**

This prospective, noncomparative, interventional case series study examined the following biomarkers: best-corrected visual acuity (BCVA), central macular thickness (CMT), central choroidal thickness (CCT), avascular area of the superficial plexus (AASP), avascular area of the deep plexus (AADP), and vessel density (VD). Data from the baseline and 4-month examinations were compared.

**Results:**

Twenty-eight eyes from 25 patients were included. Significant improvements were observed in BCVA (0.7250 ± 0.23 to 0.3957 ± 0.21; p < 0.000), CMT µm (339.04 ± 66.19 to 265.21 ± 55.75; p < 0.000), CCT µm (221.71 ± 69.69 to 209.07 ± 70.92; p < 0.000), VD (17.90 ± 7.82 to 15.35 ± 5.80; p < 0.038), AASP µm^2^ (235,374 ± 91,299 to 157,326 ± 77,815; p < 0.000) and AADP µm^2^ (996,335 ± 1,000,047 to 362,161 ± 277,225; p < 0.000). Dichotomous analysis revealed that 15 patients were responsive (53.57%), and 13 resistant (46.43%). There were no significant differences between any of the pretreatment biomarkers. In the subdivision of choroidal thickness, which ranged from 211 to 270 µm (group 3), we found greater reductions in the CCT, AADP and CD. The choroidal thickness ranged from 181 to 210 µm (group 2): BCVA and AASP exhibited the greatest reductions.

**Conclusion:**

BCVA, CMT, CCT, AASP, AADP and VD were improved after treatment. The pretreatment biomarkers did not predict treatment response between the responsive and resistant. Regarding choroidal stratification, values within the normal range of CCT showed the greatest reductions, indicating that these values may be more responsive to treatment. Notably, this is the first study to analyze biomarkers provided by SS OCT and OCTA, stratify the choroid, and perform a dichotomous analysis.

## Introduction

The Diabetic Retinopathy Clinical Research Network (DRCR.net) has been instrumental in investigating the efficacy of antiangiogenic agents in the treatment of diabetic macular edema (DME). Protocol T directly compared the efficacy of aflibercept, bevacizumab, and ranibizumab, demonstrating that aflibercept provided the greatest gains in visual acuity (VA) among patients with poorer baseline VA (≤ 20/50). Furthermore, the study reinforced the importance of individualizing treatment, highlighting that the therapeutic response may vary on the basis of the structural and functional characteristics of the patient. The five-year extension study further demonstrated that long-term outcomes were influenced by the choice of initial treatment and the need for continued therapy [1]. In this context, our study expands this perspective by using state-of-the-art imaging technology to investigate the relationship between choroidal and retinal biomarkers and the response to aflibercept. We aim to contribute to a more personalized approach in the treatment of DME.

Swept source optical coherence tomography (SS-OCT) is a novel modality with higher resolution and speed than spectral domain OCT (SD-OCT) for retinal imaging. This device (SS-OCT) has a speed of 100,000 A-scans per second and provides an 8 μm axial resolution in tissue, with a wavelength of 1050 nm. Additionally, the automatic measurement and three-dimensional (3D) reconstruction of regional images result in more reproducible and reliable measurements of choroidal thickness. The dispersion caused by the retinal pigment epithelium (RPE) is reduced by using a longer laser wavelength in SS-OCT scans, which provides a clearer boundary of the choroid-scleral interface. Therefore, swept-source (SS) technology is the best method for studying choroidal thickness (one of the objectives of this study), as its longer wavelength allows for deeper tissue penetration, enabling a more thorough analysis of these structures [2, 3].

Recently, the novel modality of optical coherence tomography angiography (OCTA), a noinvasive technique, was introduced. OCTA enables three-dimensional visualization of the choriocapillaris and retinal microvasculature by detecting blood movement within the vessels. Essentially, OCTA compares the decorrelation signal (differences in the OCT signal intensity or amplitude) between OCT B-scans taken at the same cross-section to generate a map of blood flow. SS-OCTA operates at higher wavelengths and uses ultrahigh speed, thereby improving the visualization of deeper layers [4].

Biomarkers are widely used in clinical practice for the diagnosis, prognosis, and monitoring of diseases, as well as for assessing responses to treatment. According to the National Institutes of Health, Biomarkers Definitions Group, a biomarker is defined as “a characteristic that is objectively measured and evaluated as an indicator of normal biological processes, pathogenic processes, or pharmacologic responses to a therapeutic intervention’’ [5, 6].

The identification of structural and vascular biomarkers through OCT and OCTA has played a crucial role in the evaluation of DME, enabling more accurate monitoring of disease progression and response to treatment. Different parameters obtained by these advanced technologies have been investigated as potential biomarkers for DME, including central macular thickness (CMT), choroidal vascularity index (CVI), central choroidal thickness (CCT), foveal avascular zone (FAZ), vessel density (VD), deep capillary plexus (DCP), superficial capillary plexus (SCP) and others. These metrics provide valuable information on the structural and microvascular alterations associated with DME, allowing a more personalized approach for its detection, prognosis, and therapeutic response. Furthermore, the integration of multiple biomarkers can improve the understanding of DME pathophysiology and guide more effective therapeutic strategies, thus making quantitative analysis (via OCT and OCTA) an essential tool in clinical practice and research on diabetic retinopathy (DR) [7].

Our study evaluates several biomarkers provided by SS-OCT and SS-OCTA. Additionally, this is the first study to adopt a stratified approach to choroidal thickness to identify which subgroups of patients show the greatest response to treatment with aflibercept, thus providing new perspectives on the impact of antiangiogenic therapy on the choroidal microenvironment as well as a dichotomous analysis of these biomarkers.

## Objective

The objectives of this study were to use SS technology via OCT and OCTA exams in naive patients with DME treated with a loading dose of aflibercept, over the course of a 4-month follow-up period.

The main objectives were to analyze the following:

- Key biomarkers before and after the loading dose, incluiding best-corrected visual acuity (BCVA), CMT, CCT, avascular area of the superficial plexus (AASP), avascular area of the deep plexus (AADP), and VD.
- The response to treatment of biomarkers provided by SS-OCT and SS-OCTA, which is determined by measuring the subdivision (stratification) of choroidal thickness; and.
- To perform a dichotomous analysis of the pretreatment biomarkers between the group of patients who were responsive or resistant to treatment with the loading dose of aflibercept. As a secondary objective, we assessed whether posttreatment biomarkers also showed any statistically significant differences.

## Methods

This prospective, noncomparative, interventional case series study included patients who were diagnosed with diffuse DME and were treatment-naive. The patients were recruited from Clínica Oftalmo-Retina in Presidente Prudente, SP, Brazil.

### Inclusion criteria

Patients were eligible for the study if they met the following conditions:

1. Patients aged 18 years or older were diagnosed with type 2 diabetes mellitus (DM) and diffuse DME, and had not received any prior treatment for DME (treatment-naive).
2. Three intravitreal aflibercept injections were administered by the same ophthalmologist during the study period (loading dose).
3. Comprehensive medical records containing detailed research data, including sex, age, DM duration, current medications, intraocular pressure (IOP), BCVA, SS-OCT and SS-OCTA scan results before and after treatment, were obtained.
4. BCVA between 20/25 and 20/200 was measured using the Early Treatment Diabetic Retinopathy Study (ETDRS) protocol and Snellen chart.
5. The patients provided signed informed consent, which was filled with their medical records.

### Exclusion criteria

Patients were excluded from the study on the basis of the following criteria:

1. Any prior treatment with intravitreal anti-VEGF injections was given.
2. Failure to complete the prescribed course of three intravitreal injections.
3. Presence of other significant retinal conditions, such as age-related macular degeneration, glaucoma, vitreoretinal interface disorders (epiretinal membrane, macular hole and proliferative vitreomaculopathy) or retinal dystrophies.
4. History of prior ophthalmic surgeries, including retinal detachment repair or glaucoma surgery.
5. Patients who withdrew from the treatment.

The absence of a control group in our study was justified by the nature and objectives of the paper design. This work was conceived as a prospective interventional study focused on the evaluation of retinal biomarkers in treatment-naive patients with DME before and after a loading dose. The inclusion of a control group without treatment would be incompatible with the universal ethical principles in clinical research, such as those established by the Declaration of Helsinki, which advises that all participants receive available treatment. Therefore, the absence of a control group is justified not only by the robustness of the internal comparative design, but also by the ethical obligation to provide patients with the standard treatment for DME, ensuring that the study remains aligned with the highest ethical and scientific standards.

In this study, we also conducted analyses to investigate whether the presence of certain biomarkers (variables) can indicate whether a patient will have a satisfactory response to treatment (dichotomous analysis). We divided the patients into two groups. Group 1 included responsive patients (no DME after treatment on SS-OCT: absence of intra- or subretinal fluid after the loading dose). Group 2 included resistant patients (presence of DME on SS-OCT, with intra- or subretinal fluid after the loading dose: presence of residual fluid). Therefore, we adopted a strict criterion in our study, as a patient with any amount of intra- or subretinal fluid was considered to belong to the resistant group. Thus, to analyze whether a particular biomarker/variable in the pretreatment phase could indicate whether a patient would respond to treatment with a loading dose of aflibercept, we performed a dichotomous analysis. A practical example to help understand this objective and the analyses performed is as follows. To determine whether VD could function as a risk factor for macular edema after the loading dose, we applied the statistical tests mentioned above. In general, these tests answer whether in Group 1, the variable (VD) presents higher or lower values than those in Group 2. A statistically significant would indicate that VD could predict whether the patient will respond to treatment. In other words, by looking only at the pretreatment VD values of the patients, we can determine whether a patient has a greater or lower chance of responding to treatment with the loading dose.

To study of choroidal thickness, the CCT was stratified to analyze the response of each thickness range separately.

This study adhered to the guidelines set forth in Resolution 196/96 by the National Health Council of the Ministry of Health. The research protocol was submitted for evaluation and received formal approval from the Ethics Committee of the Hospital Regional do Câncer da Santa Casa de Misericórdia de Presidente Prudente–SP (CAAE—19,386,619.1.0000.8247).

### Data collection and examination techniques

Patients who met the inclusion criteria and provided written informed consent between November 2019 and January 2022 were enrolled in the study. After being diagnosed with difusse DME and the necessary data were collected, the participants were assessed on the basis of various factors, including demographics (age, sex, duration of diabetes), medical and ocular history, diabetes classification (type 1 or 2), Diabetic Retinopathy Classification, and ophthalmologic examination. Baseline assessments included medical history, BCVA, IOP, slit lamp biomicroscopy, dilated fundus examination, 7-field color fundus photography, red-free imaging, fundus autofluorescence (FAF), fluorescein angiography (FA), SS-OCT, and SS-OCT-A. These tests were repeated four weeks after the final loading dose. BCVA was assessed using Snellen visual charts and then converted to a logarithm of the minimum angle of resolution (logMAR) units for statistical analysis. Importantly, since BCVA was converted to logMAR, a reduction in BCVA values after treatment indicates an improvement in the patient’s VA.

Retinal and choroidal images were captured using the SS-OCT and SS-OCTA (DRI-OCT Triton; Topcon–Tokyo, Japan). The images were analyzed with built-in automated layer segmentation software. Scans of 7 × 7 mm were used for SS-OCT, and 4.5 × 4.5 mm scans were used for SS-OCTA.

Retinal thickness was defined as the distance between the vitreoretinal interface and the RPE. Choroidal thickness was measured as the distance from the outer border of the RPE to the chorioscleral boundary. The automated calibration software within the SS-OCT device measured the distances between these layers. CMT and CCT were defined as the average thickness within the central 1000 µm diameter of the ETDRS grid, using the macular and choroidal thickness map provided by the SS-OCT software.

The imaging examinations were conducted in a blinded manner to reduce potential bias. All SS-OCT and SS-OCTA scans were performed by a trained technician who was unaware of the study protocol and treatment status of the patients. Two independent ophthalmologist investigators analyzed the images, and any discrepancies in measurements were resolved by a third reviewer to ensure accuracy and objectivity. Therefore they carefully examined each layer of the vitreoretinal interface, RPE, and chorio-scleral boundary to verify the segmentation accuracy. If necessary, manual adjustments were made. The SS-OCTA images included measurements of: (A) VD which are automatically provided by the OCTA software (Imaginet 6), which in this device and software corresponds to the total VD of the retina that combines the SCP and DCP. (B) FAZ of the superficial and deep plexuses (one of the advantages of this technology is that it can segment the retina). The FAZ of the superficial plexus was defined as the AASP and the FAZ of the deep plexus was defined as the AADP. These areas were measured manually by an experienced ophthalmologist and confirmed by a second specialist (the software does not automatically provide this measurement). In cases where discrepancies occurred, a third professional was consulted to ensure accurate and unbiased measurements.

To evaluate DME, SS-OCT measurements focused on identifying the presence of subretinal and/or intraretinal fluid (cysts) and detachment of the RPE and CMT above 250 µm [8].

Once DME was confirmed and intravitreal aflibercept injections were deemed necessary, patients received comprehensive information regarding the procedure, including risks, benefits, and expected visual outcomes. The study followed a structured regimen consisting of two phases: (1) an initial loading phase with three monthly intravitreal injections of aflibercept; and (2) a follow-up phase, where patients were evaluated one month after completing the loading dose (total of four months from baseline). SS-OCT and SS-OCTA examinations were performed at baseline and at the follow-up visit to assess structural and functional changes. The intravitreal injections were performed by the same experienced ophthalmologist in a surgical setting, following a standardized PRN (pro re nata) protocol**:** one injection every four weeks, totaling three injections as the loading dose. After these initial injections, patients continued to be monitored, and additional treatment was administered if DME signs persisted.

The injection process involved the application of anesthetic eye drops, 5% povidone-iodine to the conjunctival sac, and asepsis of the eyelashes, eyelids, and periorbital skin using aqueous chlorhexidine. A fenestrated drape and blepharostat were placed, and intravitreal aflibercept (0.05 ml of a 2 mg solution with a concentration of 40 mg/ml) was injected via the pars plana using a 30-gauge, 0.3 × 13 mm needle positioned 3.5 mm from the limbus. Pressure was applied to the injection site with a sterile cotton swab for one minute to prevent leakage. Postoperative care involved precription of antibiotic eye drops (0.3% tobramycin) every 6 h for 7 days.

### Statistical analysis

1. Study of patient characteristics comparisons between pre- and posttreatment: demographic characteristics such as age and sex are displayed in tables, with measures of means and standard deviations used. A Gaussian distribution test was carried out to determine whether the data followed a normal distribution. For groups within a normal distribution, the mean and standard deviation were analyzed, and Student’s t-test was employed to compare the pre- and posttreatment groups. In cases where the data did not follow a normal distribution, the Wilcoxon test was used for pre- and posttreatment comparisons.
2. Study of choroidal thickness division: a subgroup analysis was also performed by dividing the values of this pretreatment variable into four progressively increasing intervals (groups 1, 2, 3 and 4). On the basis of these levels, comparisons were made between the pre- and posttreatment values of other variables. For data with a normal distribution, the Student's t- test was applied, whereas the Wilcoxon test was used when the distribution was nonnormal.
3. Dichotomous study: To analyze the subgroup of patients who responded to the loading dose, outcome data were categorized into two groups: responsive (yes), Group 1; or resistant (no), Group 2. For continuous variables with a normal distribution, Student's t-test was used to evaluate significant differences between the responsive and resistant groups. If the data did not follow a normal distribution, the Mann–Whitney test was applied. For dichotomous nominal variables, Fisher’s exact test was used to examine associations between two variables.

To perform the statistical analysis, IBM SPSS v.24 software was used and a significance level of 5% was adopted for all analyses.

## Results

### Demographics and general results

This study included 28 eyes from 25 patients with DME treated with a loading dose of aflibercept. The mean age was 66.79 ± 10.26 years**.** The sex distribution was 48% male (12) and 52% female (13)**.** Eye laterality was 46.42% right (13) and 53.58% left (15)**.** With respect to lens status, 35.72% (10) were phakic, and 64.28% (18) were pseudophakic (Table 1).

**Table 1:** Description of clinical and personal characteristics of participants

Number of patients (N)	25
Number of eyes (N)	28
Age (years)	66.79 ± 10.26
Sex	
Male	48.00% (12)
Female	52.00% (13)
Eye	
Right	46.42% (13)
Left	53.58% (15)
Lens status	
Phakic	35.72% (10)
Pseudophakic	64.28% (18)

**Fig. 1:**
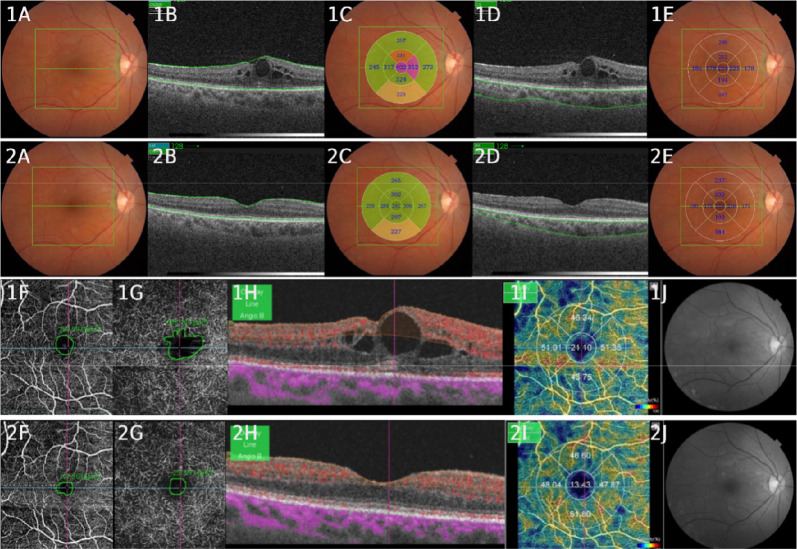
**1A** Multimodal Assessment with SS-OCT and SS-OCT-Angiography of a patient participating in this study, who received treatment with a loading dose of aflibercept (40 mg/ml) 0,05 ml/2 mg. **1A** Pretreatment: Color Retinography: presence of diabetic retinopathy (microaneurysms and cotton wool exudates). **2A** Posttreatment Color Retinography. All images that begin with number 1 correspond to pretreatment, and all images that begin with number 2 correspond to posttreatment. Image **1B** B-Scan SSOCT: delimitation of macular thickness, where we observe the presence of diabetic macular edema. 2B: Posttreatment: B-scan without macular edema. **1C** ETDRS map with central macular thickness of 402 µm, and **2C** posttreatment with central macular thickness of 241 µm. **1D** B-Scan with choroidal thickness delimitation, and **2D** choroidal thickness posttreatment. **1E** Central choroidal thickness of 224 µm pretreatment. And 2E: showing reduction to 214 µm. **1F** Avascular Area of the Superficial Plexus pretreatment: 299.597 µm^2^, and 2F: with posttreatment reduction to 197.358 µm^2^. **1G** Avascular Area of the Deep Plexus pretreatment: 655.767 µm^2^, and **2G** posttreatment with reduction to 257.871 µm^2^. **1H** B-Scan: presence of macular edema (intraretinal cysts), the red colors represent areas of greater flow according to the analysis performed by the Imaginet 6 software algorithm. **2H** Posttreatment B-Scan, absence of macular edema. **1I** Vessel Density Map = 21.10 corresponding to the central. **2I** shows decreased Central Vessel Density = 13.43 and **1J** Red Free pretreatment: presence of microaneurysms and cotton-wool exudates. **2J** Red Free posttreatment

Pre and posttreatment data were analyzed in relation to different parameters, including BCVA, CMT, CCT, IOP, VD, AASP and AADP. The results revealed significant changes in all these variables after treatment, except for IOP, which remained stable (Table 2). We obtained a statistically significant improvement in BCVA (0.7250 ± 0.23 to 0.3957 ± 0.21; p < 0,000), and significant reductions in CMT µm (339.04 ± 66.19 to 265.21 ± 55.75; p < 0,000), CCT µm (221.71 ± 69.69 to 209.07 ± 70.92; p < 0,000), CD (17.90 ± 7.82 to 15.35 ± 5.80; p = 0.038), AASP measured in µm^2^ (235,374 ± 91,299 to 157,326 ± 77,815; p < 0,000) and AADP µm^2^ (996,335 ± 1,000,047 to 362,161 ± 277,225; p < 0,000). As shown in Fig. 1, the patient who participated in this study with Multimodal Imaging Assessment with SS-OCT and SS-OCTA received treatment with a loading dose of aflibercept (40 mg/ml) 0.05 ml/2 mg: between pre- and posttreatment, we observed a reduction in CMT, CCT, AASP, AADP and VD. However, no significant changes were observed in IOP (12.68 mmHg ± 1.30 to 12.79 mmHg ± 1.44; p = 0.641). Regarding the Diabetic Retinopathy Classification by the ETDRS Classification, of the 28 eyes evaluated, 14.29% (4 eyes) were diagnosed with moderate nonproliferative DR, and 85.71% (24 eyes) were diagnosed with severe nonproliferative DR.

**Table 2:** Description of pre and posttreatment groups

Variable	Pre	Post	P-value
BCVA	0.7250 ± 0.23	0.3957 ± 0.21	0.000*
CMT (µm)	339.04 ± 66.19	265.21 ± 55.75	0.000*
CCT (µm)	221.71 ± 69.69	209.07 ± 70.92	0.000*
IOP (mmHg)	12.68 ± 1.30	12.79 ± 1.44	0.641
VD	17.90 ± 7.82	15.35 ± 5.80	0.038*
AASP (µm^2^)	235,374 ± 91,299	157,326 ± 77,815	0.000*
AADP (µm^2^)	996,335 ± 1,000,047	362,161 ± 277,225	0.000*

P-value for the Wilcoxon signed-rank test

Values less than 0.05 indicate a statistically significant difference between groups

^*^p < 0.05

BCVA: best corrected visual acuity; CMT: central macular thickness; CCT: central choroidal thickness; IOP: intraocular pressure; VD: vessel density; AASP: avascular area of the superficial plexus; AADP: avascular area of the deep plexus

### Dichotomous results

Out of a total of 28 eyes: 15 were in Group 1: Responsive (53.57%) and 13 in Group 2: Resistant (46.43%). The biomarkers/variables studied for dichotomous analysis before and after treatment were: BCVA, CMT, CCT, IOP, AASP, AADP, VD, duration of diabetes (in years), and age (Table 3); classification of diabetic retinopathy, sex of the participants, lens status (phakia or pseudophakia), type of fluid observed on SS-OCT (subretinal fluid, intraretinal fluid or pigment epithelium detachment: PED) and affected eyes: (Table 4). After analysis, none of these variables were significantly different between Group 1and Group 2 in the pretreatment analysis.

**Table 3:** Characteristics of continuous variables in the dichotomous analysis

Variable	Group-2: Resistant patients (mean ± SD)	Group-1: Responsive patients (mean ± SD)	P-value
CMT pre (µm)	357.31 ± 70.768	323.20 ± 59.827	0.108
CMT post (µm)	295.08 ± 70.071	239.33 ± 15.486	0.006*
CCT pre (µm)	222.54 ± 63.112	221.00 ± 77.157	0.955
IOP pre (µm)	12.46 ± 1.450	12.87 ± 1.187	0.294
AADP pre (µm^2^)	1,246,377.23 ± 1,285,953.068	779,632.00 ± 634,783.417	0.294
AADP post (µm^2^)	500,560.69 ± 330,168.298	242,215.20 ± 146,900.633	0.004*
AASP pre (µm^2^)	239,734.62 ± 92,549.811	231,596.27 ± 93,279.435	0.819
VD pre	19.9523 ± 10.360	16.127 ± 4.329	0.233
VD pos	18.796 ± 6.347	12.366 ± 3.152	0.002*
BCVA pre	0.678 ± 0.235	0.765 ± 0.242	0.254
BCVA pos	0.496 ± 0.260	0.308 ± 0.119	0.029*
Duration of DM (years)	14.54 ± 9.439	18.80 ± 10.213	0.294
Age (years)	68.00 ± 10.504	65.73 ± 10.299	0.57

^*^p < 0.05

BCVA: best corrected visual acuity; CMT: central macular thickness; CCT: central choroidal thickness; IOP: intraocular pressure; VD: vessel density; AASP: avascular area of the superficial plexus; AADP: avascular area of the deep plexus; DM: diabetic mellitus. Pre: pre-treatment; Post: post-treatment

**Table 4:** 2 × 2 Frequency table for analysis of dichotomous outcomes

Variable	Group- 2: resistant patients	Group-1: responsive patients	P-value
Subretinal fluid (pre)			
Presence	2	5	0.396
Absence	11	10	
Subretinal fluid (post)			
Presence	1	0	0.464
Absence	12	15	
OCT PED (pre)			
Presence	0	1	1.000
Absence	13	14	
Diabetic retinopathy classification			
Severe	12	12	0.600
Moderate	1	3	
Sex			
Male	5	7	0.718
Female	8	8	
Phakic or pseudophakic			
Pseudophakic	9	9	0.705
Phakic	4	6	
Affected eye			
Left	6	9	0.705
Right	7	6	

PED: pigment epithelium detachment; Pre: pretreatment; Post: posttreatment

On the other hand, a secondary result observed in this study was that the following posttreatment biomarkes/variables were significantly diferente (p < 0.05) between the two groups, and all presented lower values in Group 1 than in Group 2: posttreatment CMT, posttreatment AADP, posttreatment VD and posttreatment BCVA.

### Study of choroidal thickness

We subdivided the choroidal thickness into 4 groups, each with the same number of eyes (7 eyes per group). The CCT in each group was as follows: Group 1: 71 to 180 µm, Group 2: 181 to 210 µm, Group 3: 211 to 270 µm, and Group 4: 271 to 360 µm. In Group 3, we found the lowest p value (0.004) among the 4 groups of the CCT subdivision, thus indicating that Group 3 presented a greater statistically significant reduction in the CCT among all 4 groups, after the tretament (Table 5). Futhermore, there was also a more significant reduction in AADP (Table 6) and CD (Table 7) in Group 3 than in the other groups in this subdivision, after the tretament. In the Group 2, BCVA (Table 8) and AASP (Table 9) were found to have the greatest statistically significant reductions, after the treatment.

**Table 5:** Subdivided central choroidal thickness and difference between pre and posttreatment

CCT (µm)	N (eyes)	Mean reduction of CCT (µm)	P-value
71–180	7	− 9.85 ± 9.06	0.028
181–210	7	− 10.85 ± 16.74	0.137
211–270	7	− 21.42 ± 12.46	0.004*
271–360	7	− 8.42 ± 14.88	0.185

CCT: central choroidal thickness

*Smallest p value among the four groups of the choroidal thickness subdivision

**Table 6:** Reduction of avascular area of the deep plexus according to the categories of central choroidal thickness

CCT (µm)	N (eyes)	Mean reduction of AADP (µm^2^)	P-value
71–180	7	− 1,029,451 ± 1,666,052	0.018
181–210	7	− 619,497 ± 645,865	0.044
211–270	7	− 618,659 ± 455,945	0.012*
271–360	7	− 269,088 ± 473,262	0.128

CCT: central choroidal thickness; AADP: avascular area of the deep plexus

*Smallest p value among the four groups of the choroidal thickness subdivision

**Table 7:** Reduction in vessel density according to the categories of central choroidal thickness

CCT (µm)	N (eyes)	Mean Reduction in VD	P-value
71–180	7	− 0.83 ± 7.77	0.785
181–210	7	− 1.99 ± 6.89	0.473
211–270	7	− 5.52 ± 6.21	0.057*
271–360	7	− 1.85 ± 3.44	0.205

CCT: central choroidal thickness; VD: vessel density

*Smallest p value among the four groups of the choroidal thickness subdivision

**Table 8:** Reduction in BCVA according to the categories of central choroidal thickness

CCT (µm)	n (eyes)	Mean Reduction in BCVA	P-value
71–180	7	− 0.37 ± 0.28	0.018
181–210	7	− 0.29 ± 0.13	0.010*
211–270	7	− 0.18 ± 0.14	0.019
271–360	7	− 0.47 ± 0.34	0.017

CCT: central choroidal thickness; BCVA: best corrected visual acuity

*Smallest p value among the four groups of the choroidal thickness subdivision

**Table 9:** Reduction in avascular area of the superficial plexus according to the categories of central choroidal thickness

CCT (µm)	n (eyes)	Reduction in AASP (µm^2^)	P-value
71–180	7	− 45,778 ± 105,362	0.294
181–210	7	− 122,192 ± 83,131	0.008*
211–270	7	− 81,649 ± 117,461	0.116
271–360	7	− 62,574 ± 73,682	0.066

CCT: central choroidal thickness; AASP: avascular area of the superficial plexus

*Smallest p value among the four groups of the choroidal thickness subdivision

## Discussion

Glassman et al. performed a five-year extension of Protocol T and demonstrated that initial VA gains with aflibercept, bevacizumab, and ranibizumab were partially reduced over time, especially among patients who received fewer injections during follow-up. However, the study reaffirmed that aflibercept maintained a sustained superiority in improving VA in patients who had worse initial acuity (≤ 20/50), corroborating its efficacy as a therapeutic option with greater impact in these patients [1]. Notably, in this study there was a significant improvement in BCVA and a reduction in CMT with 5 intravitreal injections of aflibercept (5 months); futhermore, in our study, improvements in BCVA and a reduction in CMT also occurred, but our loading dose was 3 applications (3 months).

Several studies have shown that treatment with intravitreal injections of aflibercept reduces CMT (reduction in macular edema) and consequently improves VA. In our study, we observed the same result, similar to those reported by Brown et al., Lukic et al., and Michalska-Małecka et al. [9–11].

VD is defined as the percentage of the area occupied by vessels relative to the entire image and was one of the first metrics generated from OCTA angiograms. An early study by Hwang et al. demonstrated that VD can be used as a marker for quantifying capillary perfusion [12, 13]. With respect to total retinal VD, in our study, we observed a statistically significant decrease between baseline and after the loading dose (a finding not previously reported in the literature, when performed in the same manner as in this study: naive patients, DME patients treated with a loading dose of aflibercept in the PRN protocol and use of SS-OCT and SS-OCTA technology). This contrasts with the observations by Conti et al., who did not find a significant difference in VD between pre- and posttreatment in their study [14]. The study by Santamaria et al. (2024) demonstrated that average VD decreased after six months of anti-VEGF treatment in patients with DME, but the difference was not statistically significant [15]. Consistent with our study, Kansal et al. demonstrated that after treatment, there was a decrease in VD, but this decrease required five applications of aflibercept and occurred only in patients classified as “responders” to the treatment [16]. This highlights the complexity of studying the retina, even when advanced evaluation techniques such as SS-OCTA were used. Other factors that may influence the results include the presence of artifacts in the measurement of VD when the retina is edematous, which can lead to segmentation errors, particularly inconsistent segmentation due to the changing extent of edema with anti-VEGF therapy. Additionally, signal attenuation from macular exudation, a common finding in eyes with DME, may also confound VD measurements [17].

OCTA may help enhance the characterization of DME and its potential effects on the retinal microvasculature, particularly on the FAZ. Our data are consistent with and complement several recently published papers that also utilized OCTA to study the FAZ area in diabetic patients. These papers compared OCTA findings in patients with DR to those in healthy controls and reported that the FAZ area was significantly larger in the DR group. Additionally, Di et al. [18] reported that the FAZ area was significantly larger in the DME group than in the non-DME group in a single plexus, whereas Freiberg et al. [19] and Takase et al. [20] examined both the superficial and deep plexuses and reported more pronounced changes in the FAZ area in the deep plexus in DME group than in healthy eyes. Furthermore, Takase et al. reported a statistically significant increase in the FAZ area in diabetic eyes, regardless of the presence of DR [20]. This find suggests that changes within the FAZ may occur in the early stages of DR [21]. Additionally, our findings revealed that after loading dose treatment, the AASP decreased significantly. In contrast, Dastiridou et al. and Bush et al. demonstrated that the AASP does not decrease [22, 23].

Consisted with the finding of our study, Dastiridou et al. also demonstrated that the AADP decreased in a statistically significant manner [22]. In contrast, Bush et al. did not find a statistically significant difference between pre- and posttreatment in either the AASP or AADP [23]. Thus, the literature includes findings similar to those of our study, as well as others that present opposing results, thereby highlighting the complexity of studying these biomarkers.

In our study, a total of 28 eyes were analyzed via dichotomous analysis: 15 in Group 1: Responsive (53.57%) and 13 in Group 2: Resistant (46.43%). This result differs from that of the study published by Kansal et al., where the responsive group consisted of 20 eyes (68.96%) and the resistant group had 9 eyes (30.04%). However, in their study, 5 applications of aflibercept were performed, whereas in our study, only 3 were administered (loading dose). Another important methodological distinction between our study and that of Kansal et al. lies in the criteria used to define treatment responsiveness. In our study, a patient was classified as responsive on the basis of the complete absence of intraretinal or subretinal fluid on OCT imaging, whereas in the study of Kansal et al., responsiveness was determined on the basis solely of a reduction of ≥ 50 microns in CMT after treatment [16]. Dichotomous analysis revealed that there was no significant difference when evaluating the following pretreatment biomarkers (variables): BCVA, CMT, CCT, IOP, AASP, AADP, VD, duration of diabetes (in years), age, diabetic retinopathy classification, sex, lens status (phakia or pseudophakia), affected eye (left or right) and the type of fluid observed in SS-OCT (subretinal fluid or PED). These results suggest that, in the context of this study, these biomarkers/variables did not play a statistically significant role in indicating whether a patient would be responsive to the evaluated condition. However, other variables or factors may need to be considered for a more comprehensive and accurate understanding of DME, supporting the notion that this disease is caused by various factors (clinical, genetic, behavioral, and social). Consistently, Sorour et al. reported the same result when studying VD, concluding that this biomarker in the pretreatment phase also does not serve as a predictor of treatment response [12].

With respect to a secondary result in our study, i.e., dichotomous posttreatment results, some findings are particularly noteworthy. Certain biomarkers were significantly different (p < 0.05) between the two groups, with lower values in Group 1 than in Group 2. These were posttreatment CMT, posttreatment AADP, posttreatment VD and posttreatment BCVA. These results suggest that these variables are statistically significantly associated with Group 1. Therefore, it is possible to infer that reductions in these biomarkers occur more intensely and with significant differences when the patient is responsive to the treatment. Thus, when we have a patient who responds to treatment (group 1), there is a high chance of a joint decrease in all of the following biomarkers observed after treatment: CMT, AASP, VD and BCVA. These differences are directly related to the effects of the treatment studied. This statistically significant difference between these variables and the target condition highlights the importance of these parameters as potential biomarkers for treatment response. These findings are novel in the literature.

Normal choroidal thickness in healthy individuals generally ranges from approximately 200 to 300 µm, although these values may vary slightly depending on the population studied and the measurement techniques used. Several scientific studies have confirmed these ranges of choroidal thickness as normal in different population groups, and it is important to consider these values as a reference when interpreting clinical findings related to the choroid [24, 25]. In other studies, Campos et al. reported that the average CCT in the pretreatment phase in DME patients was 346.6 ± 75.6 µm [26], whereas in our study, it was 221.71 ± 69.69 µm. However the OCT devices used were different. In the study by Sarda et al. [27] the average CCT in the pretreatment phase was 231.7 µm, and in the posttreatment phase, it was 219.7 µm (p = 0.03). These findings are similar to ours, where we found a CCT of 221.71 µm pretreatment and 209.07 µm posttreatment. The device used in their study was the same as the one we used, but in this study 5 intravitreal injections of aflibercept or ranibizumab were used for 5 months. Another interesting result from Sarda et al. was that the CCT decreased more when aflibercept was used than when ranibizumab was used.

In the study by Dou et al. another biomarker that has also been studied in the context of DME and other retinal diseases was identified: choroidal vascularization. This biomarker is evaluated via the CVI and is obtained by binarizing images in the enhanced depth imaging (EDI) mode of OCT. Coroidal vascularization has been proposed as a more reliable biomarker to assess the response to antiangiogenic treatment than choroidal thickness alone, as it considers the proportion between the vascular and stromal components of the choroid, allowing a detailed analysis of its perfusion. The CVI has been shown to be associated with a visual response to treatment with anti-VEGF agents in patients with DME, suggesting that lower choroidal vascularization is related to a worse response to treatment. However, this study has some limitations, such as being retrospective, considering patients who gained only 5 letters of vision as responders, and having a short follow-up [28]. Thus, although the CVI may provide additional insights into the pathophysiology of DME, our choroidal thickness subdivision approach allowed us to identify subgroups of patients with greater therapeutic benefit, thus contributing to a more precise understanding of the impact of anti-VEGF therapy on the choroidal microenvironment.

The results of the CCT stratification revealed that in Group 3, there was a greater reduction in the CCT posttreatment (with the lowest p value among the four groups). These results suggest that patients with pretreatment choroidal thickness in this range may benefit more from the treatment in terms of CCT reduction than patients in other ranges. Additionally, in Group 3, we observed a greater decrease in the AADP and VD, which also suggests that patients with choroidal thickness in this range may have a more pronounced treatment response in both the deep and superficial retinal microvasculature. In Group 2 (181 to 210 µm), we observed a more significant improvement in BCVA, as well as a greater reduction in the AASP. These results suggest a complex relationship between choroidal thickness, the retinal vasculature, and visual function. In the ranges where choroidal thickness falls within normal limits (Groups 2 and 3), we observed the greatest reductions in parameters such as BCVA, AASP, CCT, AADP and VD, all of which were statistically significant. Importantly, in this category (Groups 2 and 3), the CCT was within or close to the values considered normal for choroidal thickness. This did not occur in the extremes of choroidal thickness (groups with thinner or thicker choroidal thickness): Groups 1 and 4. Thus, at the extremes of choroidal thickness, we found a less pronounced response when these parameters were evaluated. This highlights the importance of considering the subdivision of choroidal thickness as a potential biomarker in the assessment of ocular health and in understanding changes in the retinal microvasculature and visual function in response to aflibercept treatment in naive patients with DME. Consequently, the study of choroidal thickness in this paper, along with its subdivision into different ranges, allows for a more precise identification of which groups of patients may respond better to treatment in an objective manner. This highlights the importance of stratified analysis of CCT in understanding the therapeutic effects across different subpopulations, as our study is the first to subdivide choroidal thickness to assess treatment response in naive patients with DME using SS-OCT and SS-OCTA with different biomarkers, presenting novel findings not previously published in the literature.

Limitations: Our study has some limitations, such as its single-center design, small sample size, and binary classification (responsive vs. resistant) after the loading dose, which may oversimplify the response to treatment by not considering the degree of edema reduction, as we were strict in classifying patients as responders when there was a complete absence of retinal fluid (a classification that is based on significant reduction in macular thickness), and we did not use BCVA as a parameter for responsive or resistant. Despite these limitations, our findings provide valuable insights into the treatment of DME with SS-OCT and SS-OCTA in naive patients, especially regarding choroidal thickness stratification.

## Conclusion

The following biomarkers were significantly reduced after treatment with a loading dose of aflibercept: BCVA, CMT, CCT, AASP, AADP and VD.

The dichotomous analysis performed using SS-OCT and SS-OCTA did not identify any pretreatment biomarkers that could predict the response to aflibercept.

The subdivision of the CCT was proven to be an important biomarker for assessing the response to aflibercept in naive DME patients. Patients with CCTs within normal limits (Groups 2 and 3) presented significantly greater reductions in CCT, AADP, VD, BCVA, and AASP, indicating a better response to treatment than those with extreme choroidal thicknesses (Groups 1 and 4, thinner and thicker, respectively). Notably, our study is unique in that it evaluated the response of biomarkers provided by SS-OCT and SS-OCTA to treatment on the basis of the subdivision of choroidal thickness. These findings contribute to a better understanding of the relationships between choroidal characteristics and DME treatment outcomes. Overall, our study provides valuable insights into the role of choroidal thickness in predicting treatment response and highlights the potential utility of assessing choroidal thickness as an objective parameter in DME treatment.

## Data Availability

No datasets were generated or analysed during the current study.

## Abbreviations

AADP: Avascular area of the deep plexus
AASP: Avascular area of the superficial plexus
BCVA: Best-corrected visual acuity
CCT: Central choroidal thickness
CMT: Central macular thickness
CVI: Choroidal vascularity index
DCP: Deep capillary plexus
DM: Diabetes mellitus
DME: Diabetic macular edema
DR: Diabetic retinopathy
DRCR.net: Diabetic Retinopathy Clinical Research Network
EDI: Enhanced depth imaging
FA: Fluorescein angiography
FAF: Fundus autofluorescence
FAZ: Foveal avascular zone
IOP: Intraocular pressure
logMAR: Logarithm of the minimum angle of resolution
OCT: Optical coherence tomography
OCTA: Optical coherence tomography angiography
PED: Pigment epithelium detachment
PRN: Pro re nata
RPE: Retinal pigment epithelium
SCP: Superficial capillary plexus
SD-OCT: Spectral domain OCT
SS: Swept-source
SS-OCT: Swept source optical coherence tomography
SS-OCTA: Swept source optical coherence tomography angiography
VA: Visual acuity
VD: Vessel density
